# Cystic echinococcosis in Cyprus: historical retrospective and finding of 2 *Echinococcus granulosus sensu lato* species

**DOI:** 10.1017/S0031182024000520

**Published:** 2024-07

**Authors:** Azzurra Santoro, Panagiotis Konstantinou, Federica Santolamazza, Adriano Casulli

**Affiliations:** 1 WHO Collaborating Centre for the Epidemiology, Detection and Control of Cystic and Alveolar Echinococcosis, Department of Infectious Diseases, Istituto Superiore di Sanità, Rome, Italy; 2 European Union Reference Laboratory for Parasites (EURLP), Department of Infectious Diseases, Istituto Superiore di Sanità, Rome, Italy; 3 Veterinary Pathology and Parasitology Laboratory, Cyprus Veterinary Services, Ministry of Agriculture, Natural Resources and Environment, Nicosia, Cyprus

**Keywords:** Cyprus, cystic echinococcosis, *Echinococcus canadensis*, *Echinococcus granulosus sensu stricto*, genotypes G1 and G6/G7, haplogroup G7b

## Abstract

The island of Cyprus was a historical endemic area for cystic echinococcosis (CE) in the Mediterranean. During the last decades, Cyprus has been an open-air laboratory and a model for testing and implementing control measures aiming to eliminate CE as a public health problem. Despite control and surveillance measures implemented during last 50 years, molecular characterization of *Echinococcus granulosus sensu lato* specimens has been never provided. In February 2023, the carcass of a stray dog collected in the Nicosia district was examined by the Veterinary Services and found infected with *Echinococcus* spp. worms. The worms were sent to the European Union Reference Laboratory (EURLP) for species/genotype identification. The sequences analyses of *nad2* and *nad5* genes allowed us to identify the tapeworms as *Echinococcus canadensis*, genotype G7b. In November 2023, a parasitic liver cyst was observed during the post-mortem examination of a mouflon from the same area of the dog's finding. The cyst sample was also referred to EURLP for identification and comparison with tapeworms previously collected from the dog. The sequences analysis of *cox1* gene allowed to identify the cyst as *E. granulosus sensu stricto*, genotype G1. The finding of 2 different species of *E. granulosus s.l.* in a limited area raises epidemiological questions on the origin of the samples: whether distinct transmission cycles are present or a recent introduction event have occurred. From a public health perspective, it will be essential to conduct further molecular epidemiology studies to clarify the recent transmission dynamics of *Echinococcus* species in Cyprus.

## Graphical abstract

**Figure:**
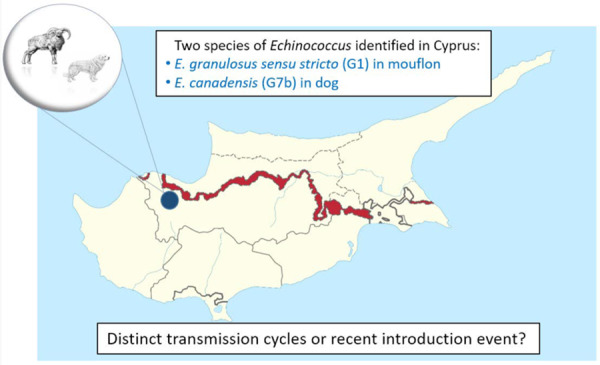

## Introduction

From an international public health prospective, cystic echinococcosis (CE) is one of the most relevant parasitic zoonotic diseases. Infection is caused by cestodes (tapeworms) belonging to *Echinococcus granulosus sensu lato* (*s.l.*) species complex. Humans can be infected by ingesting viable eggs of the parasite, which develop as cyst/s (metacestode stage) into the human body. Echinococcal cysts are space-occupying lesions mainly present in the liver and the lungs, but also in uncommon locations (Thompson, 2017; Casulli *et al*., 2023*b*). Human transmission occurs by eating contaminated food and water or by ‘hand-to-mouth’ transfer of parasitic eggs from soil, surfaces (fomites) or by direct contact with dogs (Tamarozzi *et al*., 2020). Due to its disabling and chronic condition in humans, CE is regarded as a major global public health concern and a source of economic loss (Budke *et al*., 2006; Torgerson *et al*., 2017; Casulli, 2020).

In the Mediterranean area, CE is among the most frequently diagnosed zoonoses along with rabies, leishmaniasis, brucellosis and salmonellosis (Seimenis *et al*., 2006). Despite this, CE remains an orphan parasitic disease, largely under-reported by the national health systems in Europe (Casulli *et al*., 2023*a*). CE is mainly present in rural and pastoral areas where breeding of livestock species (mainly sheep, but also goats, cattle, buffaloes, horses, camels and pigs) is practiced. Other risk factors, such as dogs free to roam, home or uncontrolled livestock slaughtering and feeding dogs with viscera, have made the Mediterranean basin a historical hotspot of CE, with many areas characterized by long-time, stable endemicity (Dakkak, 2010; Possenti *et al*., 2016; Borhani *et al*., 2020).

Although *E. granulosus s.l.* is a complex of cryptic species and genotypes, all regarded as etiologic agents of CE, the majority of human cases at both European and global level are caused by the species *E. granulosus sensu stricto* (*s.s.*) that includes the genotypes G1 and G3 (Alvarez-Rojas *et al*., 2014; Casulli *et al*., 2022). *Echinococcus granulosus s.s.* can infect several definitive and intermediate hosts, but its life cycle is most commonly sustained by a synanthropic cycle involving shepherd dogs and sheep in rural environments. The second most prevalent species in humans at both European and global level is *Echinococcus canadensis* (cluster G6/G7 genotypes), whose life cycle involves dogs as definitive hosts and swine, camels and goats as intermediate hosts (Alvarez-Rojas *et al*., 2014; Casulli *et al*., 2022). Other *E. granulosus s.l.* species causing human CE are, to a lesser extent, the ‘horse strain’ *E. equinus* (genotype G4), the ‘cattle strain’ *E. ortleppi* (genotype G5) and the ‘cervid strain’ *E. canadensis* (cluster G8/G10 genotypes) (Alvarez-Rojas *et al*., 2014; Casulli *et al*., 2022).

*Echinococcus granulosus s.s.* is acknowledged as the main driver of human CE, with similar rates in both Europe and worldwide (88.4 and 76.8% of all documented cases, respectively), while *E. canadensis* (G6/G7) rates have been found twice as high in Europe (21.7%) if compared to global estimates (11.1%) (Alvarez-Rojas *et al*., 2020; Casulli *et al*., 2022). A recent systematic review pointed out that, at European level, the geographical hotspot of human CE caused by *E. canadensis* (G6/G7) can be found in Central and Eastern Europe, although this species also circulates in other Mediterranean countries (Casulli *et al*., 2022). Animal CE caused by *E. canadensis* (G6/G7), in Central and Eastern Europe seems almost exclusively maintained by a dog-pig cycle; while in France, Greece, Italy, Portugal and Spain, *E. canadensis* G6/G7 infections are not rare also in small ruminants and wild boars (Deplazes *et al*., 2017; Sgroi *et al*., 2019; Umhang *et al*., 2019; Laurimäe *et al*., 2019*a*). Previous molecular studies conducted on *E. canadensis* confirmed the cluster G6/G7 as a distinct genotypic entity, compared to G8 and G10 (Addy *et al*., 2017). Subsequent studies have also elucidated that *E. canadensis* genotypes G6 and G7 are well distinct (Laurimäe *et al*., 2018; Laurimäe *et al*., 2019*b*). Moreover, G7 has been demonstrated to be genetically more complex than G6 with 2 distinct haplogroups (namely G7a and G7b) (Laurimäe *et al*., 2018; Laurimäe *et al*., 2019*b*). Haplogroups G7a and G7b, diversely from genotypes G6 and G7 that are largely allopatric, are eventually detected in sympatry (Laurimäe *et al*., 2019*a*; Laurimäe *et al*., 2019*b*).

The island of Cyprus is the third largest and third-most populous island in the Mediterranean Sea with an area of 9,251 km^2^ and a population density of 136 inhabitants per km^2^. Cyprus is located in the eastern Mediterranean Sea, north of Egypt, east of Greece, south of Turkey, and west of Lebanon and Syria. Livestock production accounts for 42% of the value of the total agricultural production (Papachristoforou and Markou, 2006). The cheese industry is a valuable economic sector of the island, mainly based on sheep and goat farming, corresponding to 20% to the total economic income from animal production in 2018 (Hadjipavlou *et al*., 2021). Goat and sheep farming in Cyprus is now intensive, with most of the herds with more than 100 heads. Besides sheep and goats, also pigs and cattle are raised. Cyprus mouflon (*Ovis gmelini ophion*) is endemic to the island and represents the largest wild animal species. Wild boar (*Sus scrofa*) is not currently listed among the species present in the island, despite a past attempt was conducted to introduce it from Greece for game farming (Hadjisterkotis and Heise-Pavlov, 2006).

During the past century, CE represented a severe health problem in Cyprus both for human and animals. Two different control programmes for CE, the first initiated in the 1970′s and terminated in 1985, and the second initiated in the 1990′s and still active, have significantly contributed to decrease prevalences and eliminate CE as a public health problem (Economides *et al*., 1998; Economides and Christofi, 2000). From 1997 to 2021, 57 human CE cases were officially documented in Cyprus, with an annual mean incidence of 0.22 per 100 000 inhabitants, thus ranking CE as ‘sporadic’ (Casulli *et al*., 2023*a*). In particular, official data from the Southern part of Cyprus documented 27 cases of human CE during the period 2000–2022. Most of these cases have been diagnosed in people migrated to Cyprus from the Middle East, but a smaller number of infections should be due to Greek Cypriots. Regarding animal CE, EFSA (European Food Safety Authority) officially reported positive testing cases only from mouflons in 2015 (1/23; 4.35%), 2016 (1/18; 5.56%) 2021 (2/43; 4.65%) and 2022 (1/21; 4.8%) (EFSA and ECDC, 2016, 2017, 2022, 2023).

There are no official records on molecular identification of *E. granulosus s.l.* species circulating in Cyprus, with the only exception of one *E. granulosus* G1 case found in a mouflon (unpublished data). Irrespective of the gap of knowledge on genotypes/species, since sheep and goats are the main livestock species, *E. granulosus s.s.* is expected to be the most prevalent species causing CE. In 2023, a stray dog was found infected with *Echinococcus* spp. worms in the Nicosia district. The aim of the present work was to officially document the first molecular characterization of *Echinococcus s.l.* circulating in Cyprus.

## Materials and methods

### Cases finding

**Figure 1.**
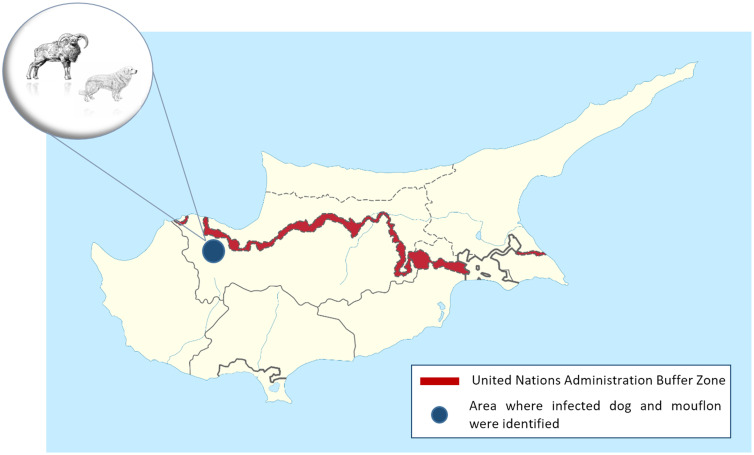
Cyprus island with the area from Nicosia district where the dog and the mouflon have been found infected by *Echinococcus granulosus sensu lato*.

In January 2023, the Game and Fauna Service collected the carcass of an adult dog from Ambelikou area (Nicosia District), close to the United Nations Buffer Zone (Fig. 1). The dog was not registered or claimed by any owner. The necropsy conducted by The Veterinary Services revealed that the dog died by illegal shot. According to the local vets, it could not be excluded that the dog was employed for shepherding. During necroscopy, the intestine was collected and tested by Sedimentation and Counting Technique (SCT) (Eckert, 2003). During the microscopic examination, *Echinococcus* spp. worms were observed in the sediment, therefore 2 pools of worms were collected, stored in ethanol 70% and sent to the European Union Reference Laboratory for Parasites (EURLP) at the Istituto Superiore di Sanità (Rome, Italy) for species identification. Later on, in November 2023, the Veterinary Services found a fertile *Echinococcus* spp. liver cyst during the necroscopy in a mouflon from Lefka area (Nicosia District). Since the positive mouflon was found few kilometres from the other finding in dog, the liver cyst was also sent to EURLP for species identification and comparison.

### Molecular analysis

Genomic DNA was extracted from a single worm from each pool and from the membrane and protoscoleces of the mouflon's cyst using the DNeasy Blood & Tissue kit (Qiagen, Valencia, CA, USA), according to the manufacturer's instructions. A method based on PCR-RFLP (targeting mitochondrial Cytochrome Oxidase I gene, *cox1*) and Multiplex PCR (targeting mitochondrial genes *Eg complex cox2*, *Eeq cox1, Ecnd G6/G7 nad1* and nuclear targets *Ecnd G8/G10 elp1*, *Eeq Cal*) (Santolamazza *et al*., 2020) was applied for *E. granulosus s.l.* species identification. To better characterize *E. canadensis* (G6/G7), fragments of the mitochondrial NAD dehydrogenase subunit 2 (*nad2*; 781 bp) and subunit 5 (*nad5*; 759 bp) were amplified by PCR and sequencing, since specific nucleotide sites of these gene fragments can be used to distinguish genotype G6 from G7, as well as distinct haplogroups within genotype G7 (Laurimäe *et al*., 2019*b*)

## Results

The PCR-RFLP and Multiplex PCR tests identified the worms as *E. canadensis*, cluster G6/G7, and the parasitic cyst as *E. granulosus s.s.*, genotypes G1-G3. Sanger sequence analysis of amplicons according to Laurimäe *et al*. (2019*b*) allowed, by specific mutations, to further type the *E. canadensis* worms as genotype G7, and more precisely as haplogroup G7b. The *nad2* and *nad5* sequences obtained were deposited in Genbank under Accession numbers OR891778-9. Sanger sequencing of amplicons obtained according to Santolamazza *et al*. (2020) typed the *E. granulosus s.s.* sample as genotype G1. To date, this finding represents the first documented molecular characterization of *E. granulosus s.l.* in Cyprus and the first detection of *E. canadensis* in this country.

## Discussion

Before 1970, CE was endemic in Cyprus and recognized as a public health problem in both animals and humans. Prior to 1970′s, animal prevalence rates of CE varied between 40% and 100% in sheep, from 20% to 50% in cattle, from 27 to 93% in goats, and from 5 to 22% in pigs (Economides *et al*., 1998). In 1972, average prevalences of 6.8% and 14% were estimated in pet and farm dogs, respectively (Economides *et al*., 1998; Christofi *et al*., 2002). In 1971, the first CE control campaign was promoted by the Department of Veterinary Services (Christofi *et al*., 2002). The control campaign of CE focused on: (1) an aggressive approach to control the dogs through registration, spaying of bitches, elimination of stray dogs and euthanasia for arecoline testing positive dogs, (2) banning of uncontrolled slaughtering of sheep and goats and (3) implementing public health education programmes (Economides and Christofi, 2000). After the creation of the United Nation Buffer Zone in 1974, the control campaign initiated in 1971 continued only in Southern part of Cyprus (Government Controlled Area; GCA), while it was suspended in the Northern part of the island (Northern Cyprus), where it was re-implemented between 1997 and 2005 and led to a decrease of the disease rates in definitive and intermediate hosts (Ruh and Taylan Özkan, 2018). Regarding the GCA, as the prevalence of CE in dogs and sheep decreased drastically, the control campaign seemed to have successfully eradicated the parasite and by 1985 the control programme was terminated (Polydorou, 1993). Indeed, human CE cases under 20 years of age have disappeared from 1990 from this area, while in Northern Cyprus the surgical cases were still present (Economides *et al*., 1998; Economides and Christofi, 2000). However, it was soon clear that despite the parasite had become more difficult to detect, it was not really disappeared from any of the 2 parts of the island. Between 1993 and 1996, *E. granulosus* was found in 82 villages from GCA in both dogs and sheep (Economides *et al*., 1998). In 1993, a new control programme started in GCA and pointed to the identification of positive animals at slaughterhouses and to trace back infections at farm level where control measures have been applied (quarantine of infected livestock and treatment of farm dogs with praziquantel within the prescribed area). The new surveillance measures also considered *Taenia hydatigena* infection as predictor of CE, since *T. hydatigena* infections are driven by similar risk factors as *E. granulosus* but with a shorter prepatent period (Economides *et al*., 1998; Economides and Christofi, 2000). The experience from Cyprus evidenced the difficulties on shifting from the initial ‘attack phase’ to the ‘consolidation phase’ to identify and eliminate the remaining sources of infection and ending with the ‘maintenance of elimination’ phase that is a long-standing surveillance to avoid CE reintroduction (as demonstrated in the Falkland Islands, Iceland, New Zealand and Tasmania) (Economides *et al*., 1998; Economides and Christofi, 2000; Craig and Larrieu, 2006).

Given these historical premises, the simultaneous detection of 2 different *E. granulosus s. l.* species in a circumscribed area is warning and raises several epidemiological questions. While *E. granulosus s.s.* finding in mouflons is not particularly surprising for a Mediterranean island, the (first) detection of *E. canadensis* in Cyprus poses some questions. It remains to be clarified if the infected dog was native to the area or imported (together with *E. canadensis*), for instance for hunting purposes. In case the dog was native, the question arises on which other hosts were sustaining *E. canadensis* transmission cycle in the island. Possible domestic intermediate hosts of *E. canadensis* (G7) are domestic pigs, goats and camels, and more rarely cattle and sheep, but there has not been any notification of infections of these species in this area. The Nicosia district, where the mouflon and the dog were found infected by *E. granulosus s.l.*, is bordering with Paphos forest where few wild boars were last spotted in 2004 (after an illegal release in Lemesos and Troodos forests in 1994 and 1996). After 2004, no further report of wild boars was documented, thus assuming the decline and extinction of this species in Cyprus (Hadjisterkotis and Heise-Pavlov, 2006). On the other hand, the infected dog could have been introduced from other countries or areas where *E. canadensis* G7 is endemic. A final possibility is that not the dog found infected in this report, but other *E. canadensis* (G7) infected dogs could have been previously imported in Cyprus from endemic areas and the transmission cycle could have just recently been established. It could be possible that dogs imported in Cyprus for hare hunting can be lost and join wild dog packs that can establish a predator–prey system with mouflons. In this scenario, the G7 intermediate host is still missing and the lack of previous molecular data makes difficult to sustain a specific hypothesis.

**Figure 2.**
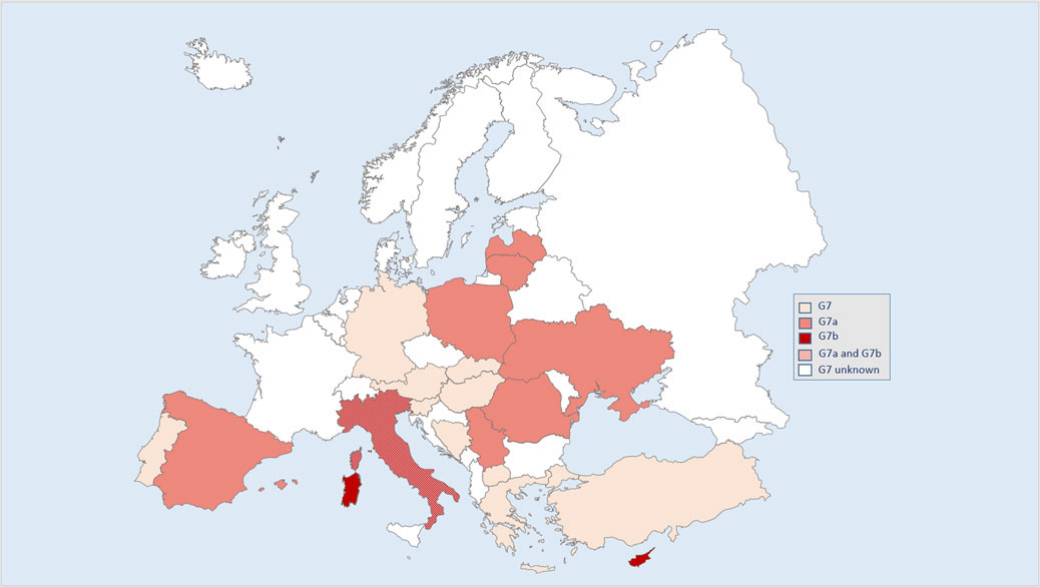
Findings of *Echinococcus canadensis* (genotype G7) in Europe. Genotype G7 can be distinguished in 2 haplogroups G7a and G7b (Laurimäe *et al*., 2018, 2019*a* and 2019*b*).

As regards Europe, it should be noticed that the genotype G7 was found in Austria (Schneider *et al*., 2010), Bosnia (Hodžić *et al*., 2022), Germany (Dinkel, *et al*., 2006), Greece (Sotiraki and Chaligiannis, 2010), Portugal (Beato *et al*., 2013), Slovakia (Šnábel *et al*., 2000), Slovenia (Šoba *et al*., 2020) and Turkey (Šnábel *et al*., 2009). Cases of G7 from Macedonia and Hungary were identified in Austria by Schneider *et al*. (2010). G7 infections were more specifically attributed to the haplogroup G7a in Spain, Italy, France (Corsica), Serbia, Romania, Poland, Lithuania (Laurimäe *et al*., 2018, 2019*a*, 2019*b*) and Latvia (Azzurra Santoro, *pers comm*). The haplogroup G7b, identified in this study, has been also found in the islands of Sardinia and Corsica (in dogs and pigs) (Laurimäe *et al*., 2018), in mainland Italy (in wild boars) (Laurimäe *et al*., 2019*a*) (Fig. 2) but also from Middle East and Central Asia (Teivi Laurimäe, *pers comm*).

Only continuous monitoring over this area in Cyprus could reveal endemic foci, if present, and if they may represent a potential threat for animal and human health. Even if the *E. canadensis* case was a singular finding, the *E. granulosus s.s.* found in the mouflon still poses a concern for the spillover of the parasite.

In conclusion, if confirmed, the established presence of both *E. granulosus s.s.* and *E. canadensis* in wild and domestic animals of Cyprus would not only corroborate the persistence of an active endemic focus but also would indicate the presence of suitable conditions to sustain differentiated life cycles, involving different hosts and transmission routes, despite control measures (consolidation phase) are still active. From a public health perspective, it is essential to promote further epidemiological studies to molecularly analyze additional samples from domestic and wild intermediate animal hosts, as well as humans, to elucidate the relative contribution of *Echinococcus* species to the infections occurring in the island. Clarifying the recent transmission dynamics of *Echinococcus* species in Cyprus would help improving the current surveillance activities to prevent the disease in both animals and humans.

## Data Availability

No additional data available.
